# Matrine suppresses thymoma stemness and apoptosis via YTH N6-methyladenosine RNA binding protein 1 and Wnt/β-catenin signaling

**DOI:** 10.1007/s13205-026-04956-z

**Published:** 2026-07-15

**Authors:** Wei Chen, Ruijian Huang, Hongqiang Chen, Ruoxin Yuan, Wu Xue

**Affiliations:** Cardiothoracic Surgery Department, Fuzhou First General Hospital Affiliated with Fujian Medical University, No.190 Dadao Road, Fuzhou, 350009 Fujian China

**Keywords:** Thymoma, Matrine, Cancer stem cells, YTHDF1, Wnt/β-catenin signaling, Apoptosis

## Abstract

Thymoma is a mediastinal tumor for which recurrence and therapeutic resistance remain clinical challenges, and cancer stem cell (CSC)-like properties may contribute to these features. This study investigated whether Matrine (Mat) affects stemness-associated features and apoptosis in EL-4-B5 cells and examined the potential involvement of YTHDF1 and Wnt/β-catenin signaling. Mat reduced cell viability and induced apoptosis, with the most evident effects observed at 100 µg/mL. Mat also reduced sphere-forming capacity and CD34 expression. Mat treatment decreased YTHDF1 expression and Wnt3a and β-catenin protein levels, whereas YTHDF1 overexpression or LiCl co-treatment partially attenuated these changes. Public-dataset analyses suggested potential clinical relevance of YTHDF1 in thymoma. Because direct m6A measurements, YTHDF1 target identification, in vivo validation, and clinical-sample validation were not performed, these findings support potential involvement of an m6A-related factor and Wnt/β-catenin signaling rather than establishing a definitive mechanism. Further validation is required before translational conclusions can be made.

**Supplementary Information:**

The online version contains supplementary material available at 10.1007/s13205-026-04956-z.

## Introduction

Thymoma is the most common primary tumor of the anterior mediastinum, originating from thymic epithelial cells and characterized by remarkable biological heterogeneity ranging from indolent growth to highly aggressive and invasive phenotypes (Rajan et al. 2024). Clinically, thymoma is frequently associated with autoimmune disorders, particularly myasthenia gravis, which significantly complicates disease management and impacts patient prognosis (Blum et al. 2020). At present, complete surgical resection remains the cornerstone of treatment for early-stage thymoma, whereas advanced or recurrent cases rely on multimodal strategies including chemotherapy, radiotherapy, and emerging targeted therapies (Tartarone et al. 2023; Muto and Okuma 2022). Despite these therapeutic advances, a substantial proportion of patients still experience recurrence and metastasis, highlighting the limitations of current treatment approaches (Luo et al. 2016). Increasing evidence suggests that tumor recurrence and therapeutic resistance are closely associated with the presence of cancer stem cells (CSCs), a subpopulation of tumor cells with self-renewal and differentiation capacities (Zhang et al. 2024). These CSCs play a crucial role in tumor initiation, progression, and resistance to conventional therapies (Falkson et al. 2023). Therefore, targeting CSC-like features in thymoma may provide a potential therapeutic direction for refractory or recurrent disease.

Matrine (Mat) is a quinolizidine alkaloid extracted from the traditional Chinese medicinal plant Sophora flavescens, which has been widely used for its anti-inflammatory and immunomodulatory properties (Li et al. 2021). In recent years, Mat has attracted increasing attention due to its broad-spectrum antitumor activities across various malignancies, including hepatocellular carcinoma, lung cancer, and breast cancer (Sun et al. 2022). Mechanistically, Mat has been reported to inhibit tumor cell proliferation, induce apoptosis and autophagy, and suppress invasion and metastasis through multiple signaling pathways (Yang et al. 2025; Zhang et al. 2019). Additionally, it can modulate the tumor immune microenvironment and enhance the sensitivity of cancer cells to chemotherapy, suggesting its potential as an adjuvant therapeutic agent (Zhang et al. 2020). Preliminary studies have also indicated that Mat exerts inhibitory effects on tumor cell growth and malignant behaviors (Xiong and Pu 2025). However, most existing studies have focused on general antitumor effects, and whether Mat regulates CSC-like properties in thymoma-related cells remains unclear (Lin et al. 2022). Thus, further research is needed to evaluate its effects on stemness-associated features in a thymoma-related model.

N6-methyladenosine (m6A) modification is the most abundant internal modification in eukaryotic mRNA and plays a critical role in post-transcriptional gene regulation (Deng et al. 2018). This dynamic and reversible process is mediated by methyltransferases, demethylases, and m6A-binding proteins, collectively regulating RNA stability, translation, and degradation (An and Duan 2022). Among these regulators, YTH N6-methyladenosine RNA binding protein 1 (YTHDF1), a key m6A reader protein, has been shown to promote the translation efficiency of target mRNAs and is frequently upregulated in various cancers (Han et al. 2019). Accumulating evidence suggests that YTHDF1 is closely associated with tumor progression, maintenance of cancer stemness, and resistance to therapy (Chai et al. 2019). Notably, YTHDF1 has been reported to regulate oncogenic signaling pathways such as Wnt/β-catenin, which is a central pathway controlling stem cell self-renewal and tumor development (Bai et al. 2019). With the rapid development of high-throughput sequencing technologies, bioinformatics analysis based on public databases such as The Cancer Genome Atlas (TCGA) has become an effective approach to identify key genes and signaling pathways involved in tumor progression (Gu et al. 2020; Zhu et al. 2018). However, whether YTHDF1 and Wnt/β-catenin signaling are associated with Mat-induced changes in thymoma-related cells remains to be determined.

Based on the above background and preliminary bioinformatics findings, our study examined whether Mat affects stemness-associated features and apoptosis in EL-4-B5 cells in association with the YTHDF1/Wnt/β-catenin axis. We first treated EL-4-B5 cells with gradient concentrations of Mat to evaluate changes in cell viability, apoptosis, and apoptosis-related protein expression. We then performed sphere formation assays and measured CD34 expression to assess stemness-associated features in EL-4-B5 cells. We further examined YTHDF1 expression and Wnt3a and β-catenin protein levels after Mat treatment. To evaluate potential involvement of this axis, we established a YTHDF1-overexpressing EL-4-B5 cell model and performed rescue experiments with LiCl. In addition, we assessed the potential clinical relevance of YTHDF1 and its association with Wnt/β-catenin-related signaling through public-dataset analyses, providing supportive contextual evidence rather than direct validation of the in vitro mechanism. Collectively, this study evaluates Mat-associated changes in stemness-related features and apoptosis and explores the potential involvement of YTHDF1/Wnt/β-catenin signaling.

## Materials and methods

### Compounds and antibodies

The murine thymic lymphoma cell line antL-4-B5 was purchased from Procell (CL-0705, Procell, Wuhan, China). Mat was purchased from APEXBIO (A3583, APEXBIO, Houston, USA). RPMI-1640 medium was purchased from Meilunbio (MA0215, Meilunbio, Dalian, China). Fetal bovine serum (FBS) was purchased from ExCell Bio (FCS500, ExCell Bio, Shanghai, China). Penicillin/Streptomycin Solution (100×, sterile) was purchased from Meilunbio (MA0110, Meilunbio, Dalian, China). The Enhanced Cell Counting Kit-8 (CCK-8) for cell proliferation and cytotoxicity assays was purchased from Meilunbio (MA0218, Meilunbio, Dalian, China). The Cell Apoptosis-Hoechst Staining Kit was purchased from Beyotime Biotechnology (C0003, Beyotime Biotechnology, Shanghai, China). Lithium chloride (molecular biology grade) was purchased from Meilunbio (MB2597, Meilunbio, Dalian, China). Western blot primary antibody dilution buffer was purchased from Meilunbio (MB9881, Meilunbio, Dalian, China). Skimmed milk powder was purchased from BBI Life Sciences (A600669-0250, BBI Life Sciences, Shanghai, China). Proteinase K Solution was purchased from Meilunbio (MA0006, Meilunbio, Dalian, China). Phosphate-buffered saline (PBS) (1×) was purchased from Meilunbio (MA0015, Meilunbio, Dalian, China). DMSO (cell culture grade) was purchased from Meilunbio (PWL064, Meilunbio, Dalian, China). Anti-CD34 antibody was purchased from BOSTER (BA3414, BOSTER, Wuhan, China). Anti-Bax antibody was purchased from BOSTER (A00183, BOSTER, Wuhan, China). Human Bcl-2 polyclonal antibody was purchased from Proteintech (12789-1-AP, Proteintech, Wuhan, China). Caspase-3/P17/P19 monoclonal antibody was purchased from Proteintech (66470-2-Ig, Proteintech, Wuhan, China). Wnt3a polyclonal antibody was purchased from Proteintech (26744-1-AP, Proteintech, Wuhan, China). β-catenin polyclonal antibody was purchased from Proteintech (51067-2-AP, Proteintech, Wuhan, China). Horseradish peroxidase (HRP)-conjugated Affinipure Goat Anti-Rabbit IgG (H + L) was purchased from Proteintech (SA00001-2, Proteintech, Wuhan, China).

### Cell culture

EL-4-B5 cells used in this study were cultured in RPMI-1640 complete medium supplemented with 10% FBS and 1% penicillin-streptomycin. The cells were maintained in a humidified incubator at 37 °C with 5% CO₂. Based on cell growth status, cells were digested and subcultured every 2–3 d using 0.25% trypsin to maintain optimal viability. As a single murine thymus-derived tumor-cell model, EL-4-B5 cells cannot fully represent the biological heterogeneity of human thymoma.

### CCK-8 assay for cell viability

This study utilized the CCK-8 assay to evaluate the effect of Mat on the viability of EL-4-B5 cells. EL-4-B5 cells were seeded in a 96-well plate at a density of 1 × 10⁴ cells per well. After routine culture for 24 h, the cells were treated with Mat at final concentrations of 0, 5, 10, 20, 50, 100, and 200 µg/mL for 48 h. Following treatment, CCK-8 solution diluted in culture medium was added to each well, and the cells were further incubated for 2 h. The absorbance of each well was then measured at 450 nm using a microplate reader. All experiments were independently repeated three times. This 48 h assay was used for initial Mat concentration screening.

### Hoechst assay for apoptosis rate

Sterile cell climbing slides (14 mm) were placed in a 24-well plate. Each well was pre-wetted with 50 µL of complete culture medium before seeding EL-4-B5 cells. After the cells adhered and stabilized, they were treated with the same Mat concentration gradient used in the CCK-8 assay for 48 h. The supernatant was discarded, and the cells were washed three times with PBS. Next, 500 µL of 4% paraformaldehyde was added to each well for fixation at 37 °C for 15 min, followed by three washes with PBS. Under light-protected conditions, 200 µL of Hoechst 33,342 staining solution was added to each well, and the cells were incubated at room temperature for 25 min, followed by five washes with PBS. The cell climbing slides were mounted with an anti-fade mounting medium and observed under an inverted fluorescence microscope. For each group, three random fields of view were selected for image acquisition and analysis.

### YTHDF1 overexpression, experimental groups, and treatment timeline

To investigate the potential involvement of YTHDF1 in Mat-associated effects, stable EL-4-B5 cell lines overexpressing YTHDF1 and corresponding negative-control cells were established using a lentivirus-mediated method. Specifically, the full-length coding sequence of mouse YTHDF1 was cloned into the pLVX-Puro lentiviral vector, and the empty pLVX-Puro vector was used as the negative control. Lentiviral particles were produced in 293 T cells by co-transfection with the packaging plasmids psPAX2 and pMD2.G. EL-4-B5 cells in the logarithmic growth phase were infected at a multiplicity of infection (MOI) of 20 in the presence of 8 µg/mL polybrene. At 48 h after infection, cells were selected with 2 µg/mL puromycin for 7 d and maintained with 1 µg/mL puromycin. Overexpression efficiency was verified by qPCR and Western blot. Subsequently, stable cells and wild-type cells in the logarithmic growth phase, with a confluence of approximately 50%–70%, were used for experimental grouping. The groups included: a control group without drug treatment; a Mat group treated with 100 µg/mL Mat for 24 h; a Mat + LiCl group pretreated with 10 mM LiCl for 24 h before treatment with 100 µg/mL Mat; a Mat + YTHDF1 NC group treated with 100 µg/mL Mat for 24 h; and a Mat + YTHDF1 OE group treated with 100 µg/mL Mat for 24 h. The 100 µg/mL Mat concentration was selected because it produced the strongest combined effects on cell viability and apoptosis in the initial screening. A 24 h endpoint was selected for mechanistic and rescue experiments to assess molecular and phenotypic changes before prolonged exposure-associated loss of viability became predominant. Cells were collected after the specified treatment period for subsequent assays.

### Sphere formation assay

To evaluate the effects of the aforementioned treatment groups on stemness-associated features of EL-4-B5 cells, a sphere formation assay was conducted. This assay was based on the stable cell models and grouping scheme established in the section “YTHDF1 Overexpression, Experimental Groups, and Treatment Timeline,” including the Control group, Mat group, Mat + LiCl group, Mat + YTHDF1 NC group, and Mat + YTHDF1 OE group. Cells from each group after the corresponding treatment were collected, centrifuged, and washed with pre-cooled PBS. The cells were then resuspended in serum-free Dulbecco’s Modified Eagle Medium/Nutrient Mixture F-12 (DMEM/F12) medium (11320-033, Gibco, Grand Island, NY, USA) supplemented with 20 ng/mL epidermal growth factor (EGF) (AF-100-15, PeproTech, Rocky Hill, NJ, USA), 10 ng/mL bFGF (AF-100-18B, PeproTech, Rocky Hill, NJ, USA), 1 × B27 supplement (17504-044, Gibco, Grand Island, NY, USA), and 1% penicillin-streptomycin (SV30010, HyClone, Logan, UT, USA). Cells were seeded into a 96-well ultra-low attachment plate at a density of 1 × 10³ cells per well in a volume of 200 µL, with three replicate wells per group. The peripheral wells of the plate were filled with PBS to minimize evaporation. The seeded plate was incubated at 37 °C with 5% CO₂ for 7 d, with daily observation of sphere formation under an inverted microscope. After the culture period, three random non-overlapping fields were captured from each replicate well using an Eclipse TS2 inverted microscope (Nikon, Tokyo, Japan), giving nine analyzed fields per group. Images were analyzed using ImageJ software (version 1.54 g) after spatial calibration. Spheres with a diameter ≥ 50 μm were counted, and sphere area percentage was calculated as the total projected area of qualifying spheres divided by the total analyzed image area × 100%. The mean value of the three fields from each replicate well was used for statistical analysis.

### Western blot analysis for apoptosis markers, stemness markers, and Wnt/β-catenin pathway proteins

Cells from each group treated according to the section “YTHDF1 Overexpression, Experimental Groups, and Treatment Timeline” were collected at designated time points. The culture medium was discarded, and the cells were washed twice with pre-cooled PBS. An appropriate volume of RIPA lysis buffer containing protease inhibitor (No. P0013B, Beyotime, China) was added to each cell sample, followed by lysis on ice for 30 min. Subsequently, the lysates were centrifuged at 4 °C and 15,000 × g for 10 min, and the supernatant was collected as the total protein solution. Protein concentration was determined using the Bicinchoninic Acid method. Equal amounts of total protein were separated by 10% SDS-polyacrylamide gel electrophoresis and then transferred onto a polyvinylidene fluoride membrane using the wet transfer method. After transfer, the membrane was blocked with 5% skimmed milk at room temperature for 2 h. Following Tris-Buffered Saline with Tween-20 (TBST) washes, the membrane was incubated with primary antibodies against CD34, Bax, Bcl-2, Caspase-3, Wnt3a, β-catenin, or β-actin at 4 °C overnight. The next day, after thorough washing with TBST, the membrane was incubated with corresponding HRP-conjugated secondary antibodies at room temperature for 2 h. After washing, signals were developed using enhanced chemiluminescence reagent, captured by a chemiluminescence imaging system, and analyzed with ImageJ software. Band intensities of target proteins were normalized to β-actin in the corresponding samples, and densitometric results were obtained from three independent experiments.

### Quantitative real-time PCR analysis of YTHDF1 expression

Total RNA was extracted from the five groups of cells treated as described in the “YTHDF1 Overexpression, Experimental Groups, and Treatment Timeline” section using TRIzol reagent, and subsequently reverreal-timeal-timbed into cDNA using a commercial reverse transcription kit. Quantitative real-time PCR (qPCR) was performed using a SYBR Green-based detection system in a total reaction volume of 20 µL containing 10 µL of 2× SYBR Green Master Mix, 0.4 µL of forward primer (10 µM), 0.4 µL of reverse primer (10 µM), 2 µL of diluted cDNA template, and 7.2 µL of nureal-timase-free water, with GAPDH serving as the internal reference gene. The primer sequences were as follows: GAPDH (mouse): forward 5′-TGGAAAGCTGTGGCGTGATG-3′, reverse 5′-TACTTGGCAGGTTTCTCCAGG-3′;

YTHDF1 (mouse): forward 5′-CACACAACCTCTATCTTTGACGA-3′, reverse 5′-ACTGGCTTGTTCTTATTGTTTGT-3′ (Wang et al. 2024). Each sample was analyzed in triplicate. The cycling program was as follows: initial denaturation at 95 °C for 30 s; 40 cycles of denaturation at 95 °C for 5 s and annealing/extension at 60 °C for 30 s; followed by melting-curve analysis from 65 °C to 95 °C. The relative mRNA expression level of YTHDF1 was calculated using the 2^(-ΔΔCt) method, and differences among groups were statistically analyzed.

### Statistical analysis

All experiments were independently repeated three times (or as indicated in the figure legends). Experimental data are expressed as mean ± standard deviation (x̄ ± s). Differences between groups were analyzed using one-way analysis of variance (ANOVA) or t-test, as appropriate to the experimental design. All statistical analyses were performed using GraphPad Prism software (version 10.1.2). A P-value < 0.05 and P-value < 0.01 were considered statistically significant and highly statistically significant, respectively.

### Bioinformatics analysis based on TCGA-THYM and genotype-tissue expression (GTEx) data

Uniformly processed RNA-seq expression data were obtained from the UCSC Xena TCGA/GTEx RNA-seq compendium. The expression comparison included 120 TCGA-THYM tumor samples and 339 Genotype-Tissue Expression (GTEx) normal thymus samples. Corresponding clinical information for TCGA-THYM patients was obtained from TCGA, and 119 patients with complete overall survival information were included in the survival analysis. For survival analysis, patients were divided into high- and low-YTHDF1 expression groups based on the median expression value. Kaplan–Meier survival curves were generated to evaluate overall survival, and statistical significance was assessed using the log-rank test. Univariate Cox proportional hazards regression analysis was performed to evaluate the prognostic association of YTHDF1, and hazard ratios with 95% confidence intervals were calculated. Gene set enrichment analysis (GSEA) was conducted to explore signaling pathways associated with YTHDF1 expression. Patients were stratified into high- and low-expression groups, and enrichment of the Wnt/β-catenin signaling pathway was analyzed using Kyoto Encyclopedia of Genes and Genomes gene sets. All statistical analyses and visualizations were performed using R software (version 4.3.2) with packages including ggplot2, survival, and clusterProfiler. A two-sided *P* < 0.05 was considered statistically significant. These analyses were interpreted as supportive evidence for potential clinical relevance rather than direct validation of the in vitro mechanism.

## Results

### Mat reduces cell viability and promotes apoptosis

**Fig. 1:**
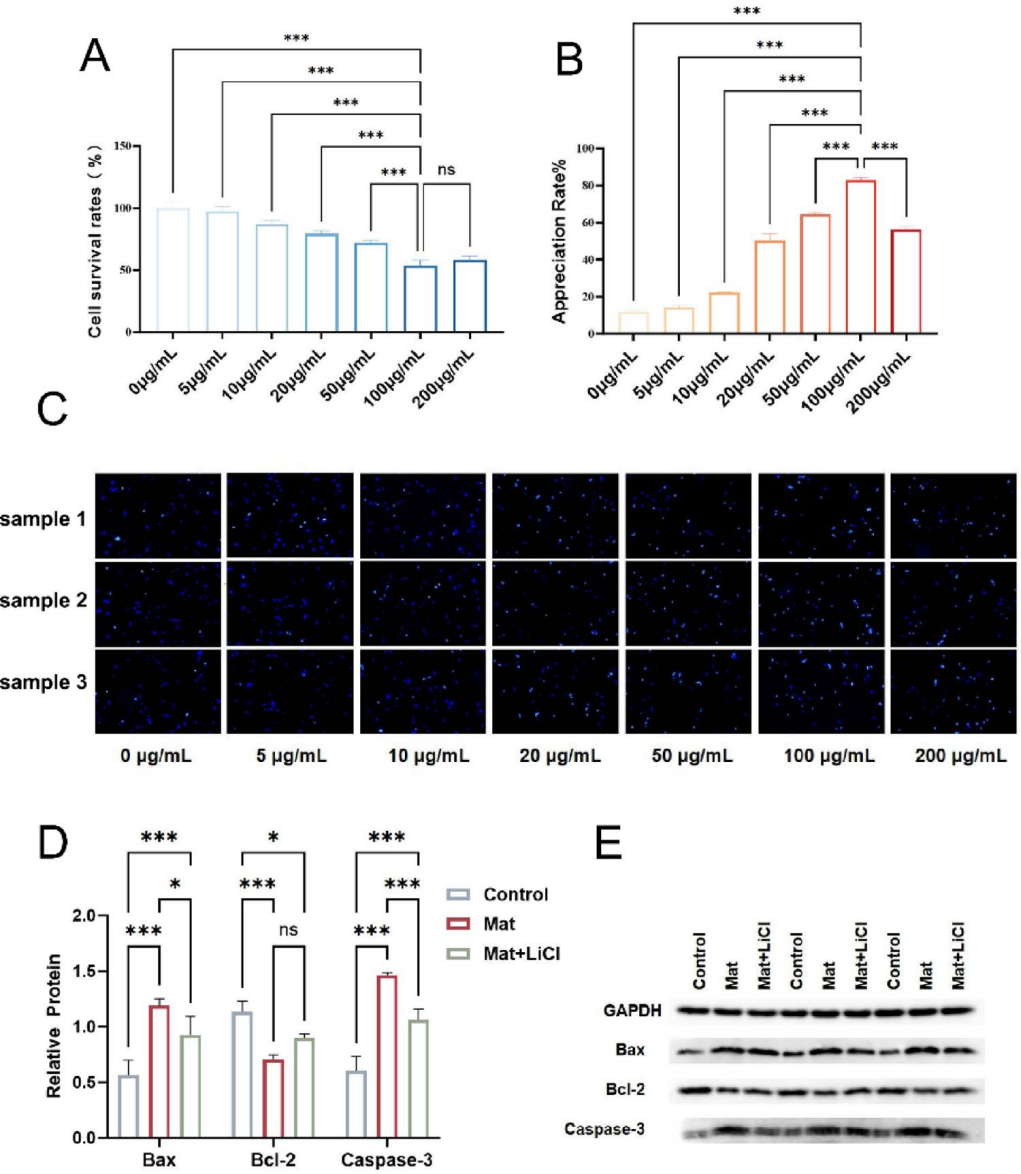
Mat reduces cell viability and induces apoptosis in EL-4-B5 cells. **A **Cell viability measured by the CCK-8 assay after treatment with different concentrations of Mat **B** Apoptosis rate detected by Hoechst 33,342 staining **C** Representative fluorescence images of Hoechst 33,342 staining (scale bar: 50 μm). Normal nuclei appear homogeneous light blue, while apoptotic nuclei are bright blue with condensed or fragmented morphology **D** Western blot analysis of apoptosis-related proteins (Bax, Bcl-2, and Caspase-3) expression in the control, Mat, and Mat + LiCl groups **E** Statistical analysis of the relative quantitative expression of each protein. Relative protein expression levels were determined using ImageJ software. Data are presented as mean ± SD (*n* = 3). ns, not significant; *P < 0.05, **P < 0.01, ***P < 0.001, and ****P < 0.0001

CCK-8 asreduces cell viability and promotes apoptosisy results (Fig. 1A) showed that Mat reduced EL-4-B5 cell viability with a concentration-dependent trend from 5 to 100 µg/mL. Within this range, cell survival rates significantly decreased with increasing concentration (*P* < 0.001), with the strongest inhibitory effect observed at 100 µg/mL. At 200 µg/mL, cell viability slightly recovered compared to the 100 µg/mL group. Hoechst staining results (Fig. 1B, C) demonstrated that Mat induced apoptosis, with the apoptosis rate first increasing and then decreasing as the concentration rose. The highest apoptosis rate occurred in the 100 µg/mL group (*P* < 0.001), while it significantly declined in the 200 µg/mL group (*P* < 0.001). Western blot analysis further confirmed that 100 µg/mL Mat significantly upregulated the expression of pro-apoptotic proteins Bax and Caspase-3 (*P* < 0.001) and downregulated the expression of anti-apoptotic protein Bcl-2 (*P* < 0.05) (Fig. 1D, E). The original Western blot images are presented in Fig. S1. Because 100 µg/mL produced the strongest combined reduction in viability and increase in apoptosis, this concentration was selected for subsequent mechanistic experiments. The basis for the non-monotonic response at 200 µg/mL remains to be investigated. 

### Mat reduces sphere formation and CD34 expression

**Fig. 2:**
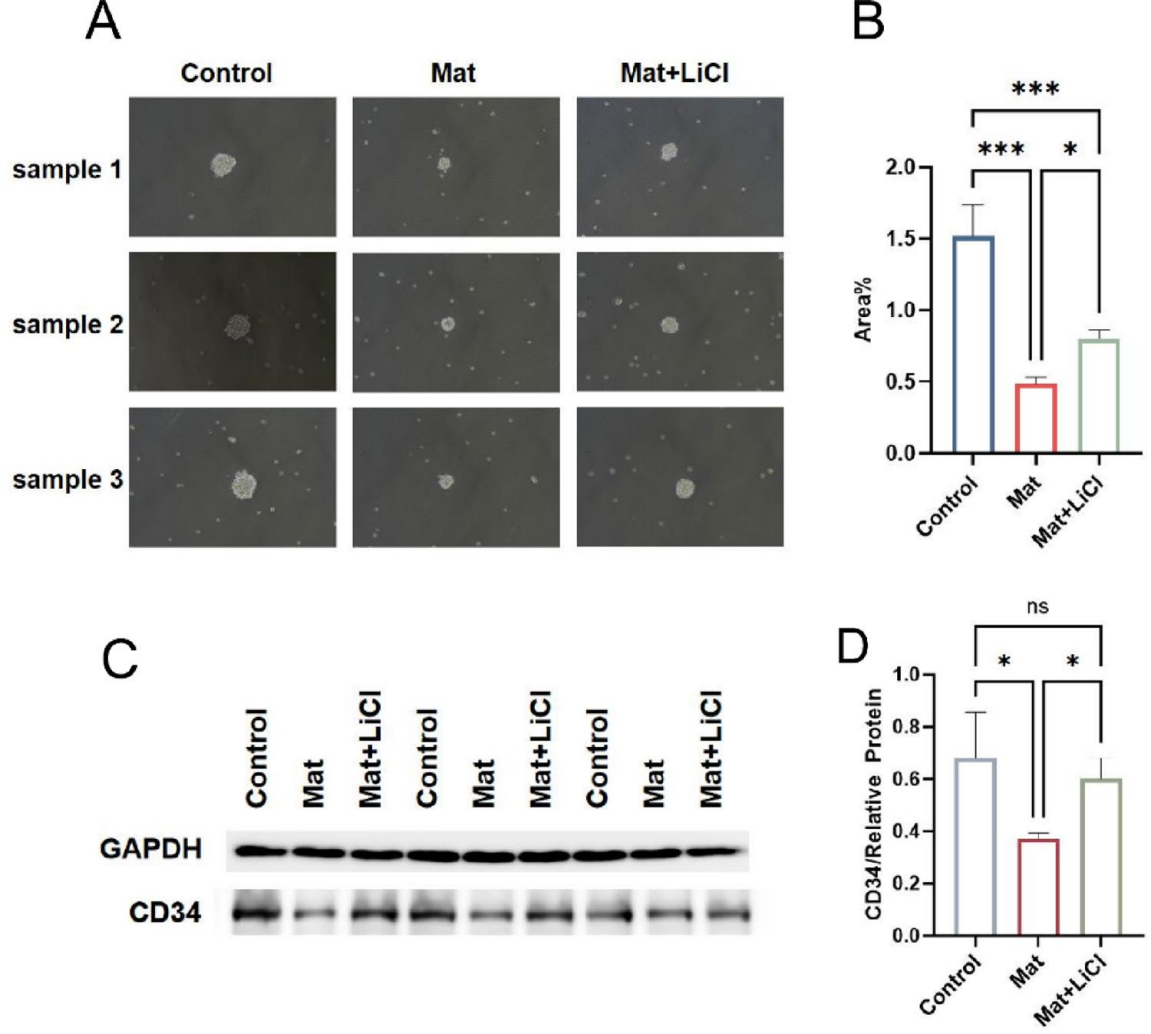
Effects of Mat on sphere formation and CD34 expression. **A** Representative microscopic images from the sphere formation assay **B** Quantitative analysis of sphere area percentage in each group. Sphere area percentage was defined as the total projected area of qualifying spheres divided by the total analyzed image area × 100% **C** Western blot detection of the stemness marker CD34 expression in the Control, Mat, and Mat + LiCl groups **D **Quantitative statistics of the relative expression level of CD34 protein. Relative protein expression levels were determined using ImageJ software. Data are presented as mean ± SD (*n* = 3). *P < 0.05, **P < 0.01, ***P < 0.001; ns indicates no significant difference

To evaluate stemness-associated features, sphere formation and CD34 protein expression were assessed after Mat treatment. Mat treatment significantly reduced both sphere number and sphere area percentage compared with the control group (Fig. 2A, B; *P* < 0.001). Western blot analysis further showed reduced CD34 protein expression after Mat treatment (Fig. 2C, D; *P* < 0.05). The original Western blot images are presented in Fig.S2. These findings are consistent with reduced stemness-associated features after Mat treatment; however, they should be interpreted together with the concurrent reduction in cell viability.

### YTHDF1 overexpression partially attenuates Mat-associated changes

**Fig. 3:**
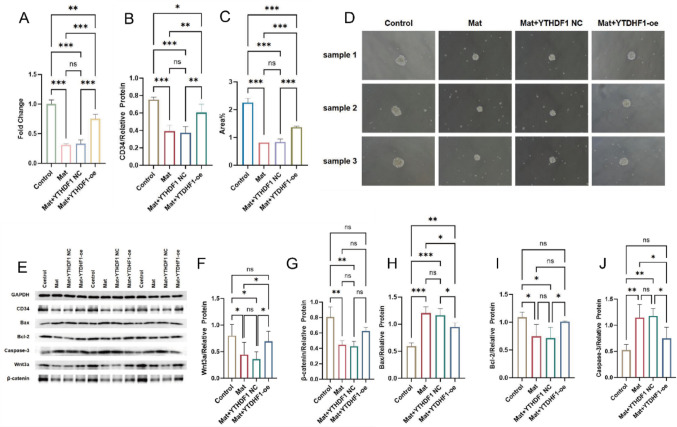
YTHDF1 overexpression partially attenuates Mat-associated changes. **A** Relative mRNA expression level of YTHDF1 detected by qPCR in the Control, Mat, and Mat + YTHDF1 OE groups **B** Western blot analysis of the stemness marker CD34 expression in cells from each group **C** Quantitative analysis of sphere area percentage in each group from the sphere formation assay. Sphere area percentage was defined as the total projected area of qualifying spheres divided by the total analyzed image area × 100% **D** Representative microscopic images from the sphere formation assay **E**. Western blot analysis of Wnt3a, β-catenin, and apoptosis-related proteins (Bax, Bcl-2, Caspase-3) expression in cells from each group **F**-**G** Quantitative analysis of the relative expression levels of Wnt3a and β-catenin proteins **H-J** Quantitative analysis of the relative expression levels of Bax, Bcl-2, and Caspase-3 proteins. Relative expression levels were determined using ImageJ software. Data are presented as mean ± SD (*n* = 3). *P < 0.05, **P < 0.01, ***P < 0.001; ns indicates no significant difference

To assess whether YTHDF1 is associateovereith Mat-induced changes, YTHDF1 expression and related readouts were evaluated in Mat-treated cells and in cells subjected to YTHDF1 overexpression. qPCR results (Fig. 3A) showed that compared to the control group, Mat treatment significantly reduced YTHDF1 mRNA expression levels (*P* < 0.001). Overexpression of YTHDF1 on the basis of Mat treatment effectively restored its expression compared to the Mat-alone group (*P* < 0.001), though it remained lower than the control level. This indicates that Mat downregulates YTHDF1 expression, an effect that can be partially reversed by exogenous overexpression. Further analysis combining stemness phenotype and molecular marker detection revealed that Mat treatment downregulated CD34 protein expression while reducing both the percentage of sphere area and the number of spheres (Fig. 3B-D), consistent with reduced stemness-associated features.

Compared with Mat treatment alone, YTHDF1 overexpression partially restored CD34 protein levels and sphere area percentage (Fig. 3B, C). Regarding apoptosis-related readouts, Western blot analysis (Fig. 3E-J) showed that Mat treatment increased Bax and Caspase-3 expression and decreased Bcl-2 expression. The original Western blot images are presented in Fig. S3. Following YTHDF1 overexpression, Bax and Caspase-3 levels decreased, while Bcl-2 expression partially recovered. Mat treatment also reduced Wnt3a and β-catenin protein expression, whereas YTHDF1 overexpression partially restored the expression levels of these two proteins. These results show that YTHDF1 overexpression partially attenuated several Mat-associated changes, but they do not establish direct m6A-dependent regulation or a unique downstream mechanism.

### LiCl co-treatment partially attenuates Mat-associated changes

**Fig. 4:**
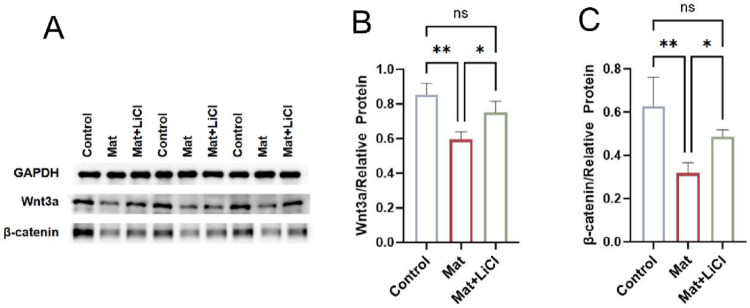
LiCl co-treatment partially attenuates Mat-associated changes in Wnt3a and β-catenin expression. **A** Western blot analysis of Wnt3a and β-catenin expression in the Control, Mat, and Mat + LiCl groups **B** Quantitative analysis of the relative expression level of Wnt3a protein **C** Quantitative analysis of the relative expression level of β-catenin protein. Relative protein expression levels were determined using ImageJ software. Data are presented as mean ± SD (*n* = 3). *P < 0.05, **P < 0.01; ns indicates no significant difference

To assess whether Wnt/β-catenin signaling may be involved in Mat-associated changes, LiCl co-treatment was used as a pharmacological pathway-activation approach. Mat treatment significantly reduced the expression levels of Wnt3a and β-catenin compared with the control group (*P* < 0.01). Further combined treatment with LiCl revealed that LiCl could partially alleviate the inhibitory effect of Mat on this pathway, as the protein expression levels of Wnt3a and β-catenin showed some recovery compared to the Mat-only group (Fig. 4A-C; *P* < 0.05). The original Western blot images are presented in Fig. S4.

LiCl co-treatment partially attenuated Mat-associated changes in apoptosis-related proteins, sphere-forming capacity, and CD34 expression (Figs. 1D and E and 2A–D). These rescue findings support potential involvement of Wnt/β-catenin signaling in Mat-associated phenotypes. However, because LiCl is not entirely pathway-specific, this experiment does not by itself establish a specific causal mechanism.

### Public-dataset analysis of the potential clinical relevance of YTHDF1 based on TCGA-THYM and GTEx data

**Fig. 5:**
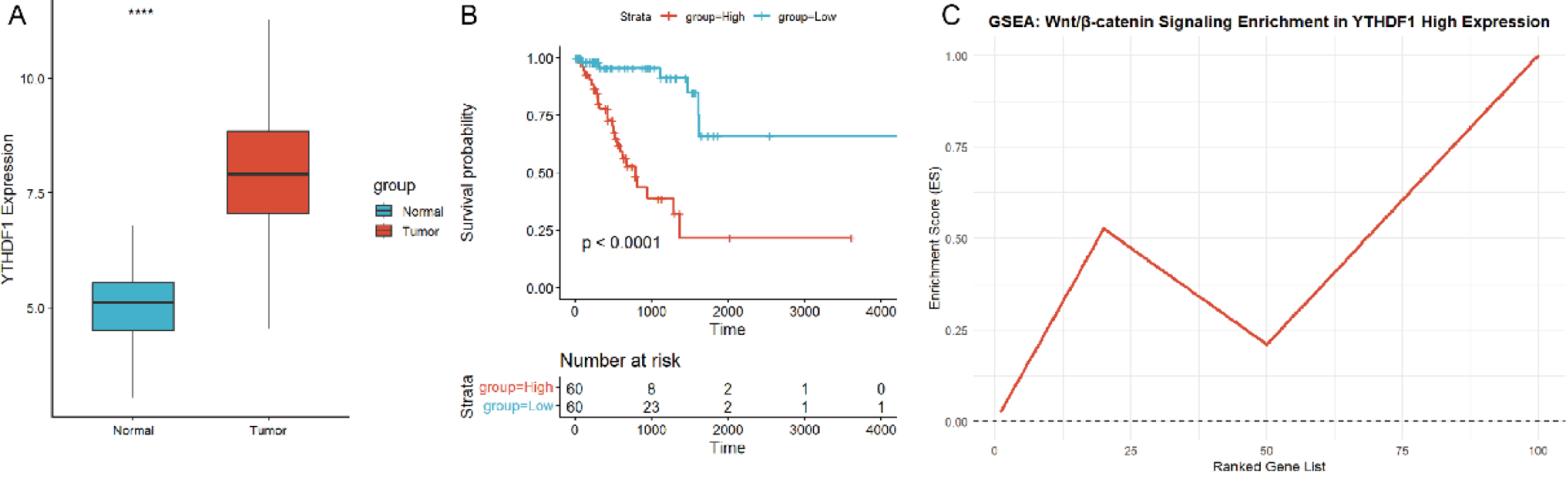
Potential clinical relevance and pathway association of YTHDF1 based on TCGA-THYM and GTEx data **A **Box plot comparing the mRNA expression levels of YTHDF1 between TCGA-THYM tumor samples and GTEx normal thymus samples. **** indicates a highly statistically significant difference in expression between the two groups (*P* < 0.0001) **B** Kaplan–Meier survival curves for overall survival of thymoma patients, stratified into high and low YTHDF1 expression groups based on the median expression value. The log-rank test was used to evaluate the statistical difference in survival between the two groups (*P* < 0.0001). The number at risk table below the curve presents the number of surviving patients in each group at the corresponding time points **C** GSEA plot showing the significant enrichment of the Wnt/β-catenin signaling pathway in the YTHDF1 high expression group of thymoma patients. ES: Enrichment Score

Public-dataset analyses were performed to assess the potential clinical relevance of YTHDF1. YTHDF1 expression was higher in TCGA-THYM tumor samples than in GTEx normal thymus samples (Fig. 5A). Kaplan–Meier survival analysis demonstrated that patients with high YTHDF1 expression had significantly poorer overall survival than those with low expression levels (Fig. 5B). To gain insight into the potential mechanisms associated with YTHDF1, GSEA was conducted. The results revealed significant enrichment of the Wnt/β-catenin signaling pathway in the YTHDF1 high-expression group (Fig. 5C). Moreover, univariate Cox regression analysis indicated that elevated YTHDF1 expression was associated with unfavorable prognosis, suggesting its potential prognostic relevance. Collectively, these findings provide supportive clinical-context evidence for YTHDF1 but do not directly validate the in vitro mechanism or establish YTHDF1 as an independent prognostic factor.

## Discussion

In this study, we investigated the effects of Mat on EL-4-B5 cell behavior, with a focus on stemness-associated features and apoptosis-related readouts. Through cell viability, apoptosis, sphere formation, and protein-expression analyses, we found that Mat reduced cell viability, increased apoptosis-associated readouts, and decreased sphere formation and CD34 expression. Mat treatment was associated with reduced YTHDF1 expression and reduced Wnt/β-catenin pathway-related protein levels, while functional rescue experiments showed that YTHDF1 overexpression or LiCl co-treatment partially attenuated several Mat-associated changes. In addition, public-dataset analysis supported the potential clinical relevance of YTHDF1 in thymoma. Overall, our study provides evidence for potential involvement of an m6A-related factor in Mat-associated phenotypes, although further in vivo and clinical validation is still required.

This study incorporates the m6A reader protein YTHDF1 into the investigation of stemness-associated features in a thymus-derived tumor-cell model, highlighting it as a potential factor associated with Mat-induced phenotypes. This observation suggests a possible m6A-related component in the regulation of tumor stemness (Shi et al. 2019). The role of m6A modifications in cancer biology has garnered increasing attention, particularly in influencing gene expression at the post-transcriptional level (Huang et al. 2020). Beyond these broad regulatory functions, m6A has also been implicated in the metabolic reprogramming of cancer cells (Li et al. 2020). At the effector level, YTHDF1 has been shown to enhance the translation efficiency of oncogenic transcripts, thereby promoting tumorigenesis (Pi et al. 2021). In addition, accumulating evidence suggests that RNA methylation is closely associated with the maintenance of CSC phenotypes (Cui et al. 2017). Integrative analyses across multiple cancer types further support the widespread involvement of m6A regulators in tumor progression (Chai et al. 2019). In the present study, YTHDF1 expression was associated with Mat-induced changes in stemness-related readouts and Wnt/β-catenin-related proteins in EL-4-B5 cells. Direct m6A measurements and identification of YTHDF1-bound target transcripts were not performed; therefore, direct epitranscriptomic regulation cannot be concluded.

These results suggest that Mat reduces stemness-associated features in association with Wnt/β-catenin signaling. This interpretation extends beyond Mat’s conventional roles in inhibiting proliferation or inducing apoptosis. This finding highlights a previously underappreciated functional dimension of Mat in regulating cancer cell plasticity and stem-like characteristics (Nguyen et al. 2012). The pharmacological versatility of Mat has been well documented in a variety of disease models. Its antitumor activity has been linked to the regulation of multiple signaling pathways involved in inflammation, proliferation, and malignant progression (Shen et al. 2024). Notably, targeting Wnt signaling with natural compounds has emerged as a promising therapeutic strategy in cancer treatment (Zheng et al. 2025). In addition to pathway modulation, Mat has also been associated with the induction of apoptosis and autophagy in several cancer models (Zhang et al. 2021). More recent evidence further suggests that natural compounds can directly influence CSC properties (Debnath and Kundu 2025). Compared with these studies, the present work integrates Mat, Wnt signaling, and tumor stemness within a unified framework. It also introduces YTHDF1 as a potential upstream regulatory factor, providing a more mechanistically coherent interpretation.

Several limitations should be acknowledged. First, the experiments were primarily conducted in vitro using only the murine thymic lymphoma cell line EL-4-B5, and the results cannot fully represent the heterogeneity or clinical biology of human thymoma. Second, the basis for the non-monotonic response observed at 200 µg/mL Mat remains unclear and requires further investigation. Third, the downstream regulatory network of YTHDF1 in EL-4-B5 cells has not been fully elucidated, and direct m6A measurements, identification of YTHDF1-bound target transcripts, and pathway-specific validation were not performed. Fourth, in vivo efficacy, pharmacokinetics, safety, and clinical-sample validation remain necessary before translational conclusions can be drawn. Despite these limitations, the present study provides preliminary evidence that Mat-associated changes in stemness-related features may involve the YTHDF1/Wnt/β-catenin axis. These findings provide a basis for further investigation in thymoma-related models.

## Conclusion

In summary, Mat reduced viability, decreased stemness-associated features, and promoted apoptosis-associated changes in EL-4-B5 cells. Mat treatment was accompanied by decreased YTHDF1 expression and reduced Wnt/β-catenin pathway-related protein expression. Functional rescue experiments further suggest that YTHDF1 overexpression or pharmacological activation of Wnt/β-catenin signaling can partially attenuate Mat-associated changes in stemness- and apoptosis-related readouts. These findings suggest potential involvement of the YTHDF1/Wnt/β-catenin axis in Mat-associated phenotypes. Public-dataset analyses further support the potential clinical relevance of YTHDF1. However, direct molecular mechanisms, pathway specificity, in vivo effects, and clinical applicability require further validation. Mat therefore warrants further investigation in thymoma-related models rather than being considered an established therapeutic strategy.

## Supplementary Information

Below is the link to the electronic supplementary material.

Supplementary Material 1

## Data Availability

The experimental data used to support the findings of this study are available from the corresponding author upon request. The TCGA-THYM and GTEx datasets analyzed in this study are publicly available through the UCSC Xena data resource. The experimental data supporting the findings of this study are available from the corresponding author upon reasonable request.
