# Supplementary material for: Matrine suppresses thymoma stemness and apoptosis via YTH N6-methyladenosine RNA binding protein 1 and Wnt/β-catenin signaling
Source: 3 Biotech. 2026 Jul 15;16(8):331. doi: 10.1007/s13205-026-04956-z (PMC13369092; doi:10.1007/s13205-026-04956-z)
Supplement: Supplementary file 1 — Supplementary Material 1 [file 13205_2026_4956_MOESM2_ESM.docx]

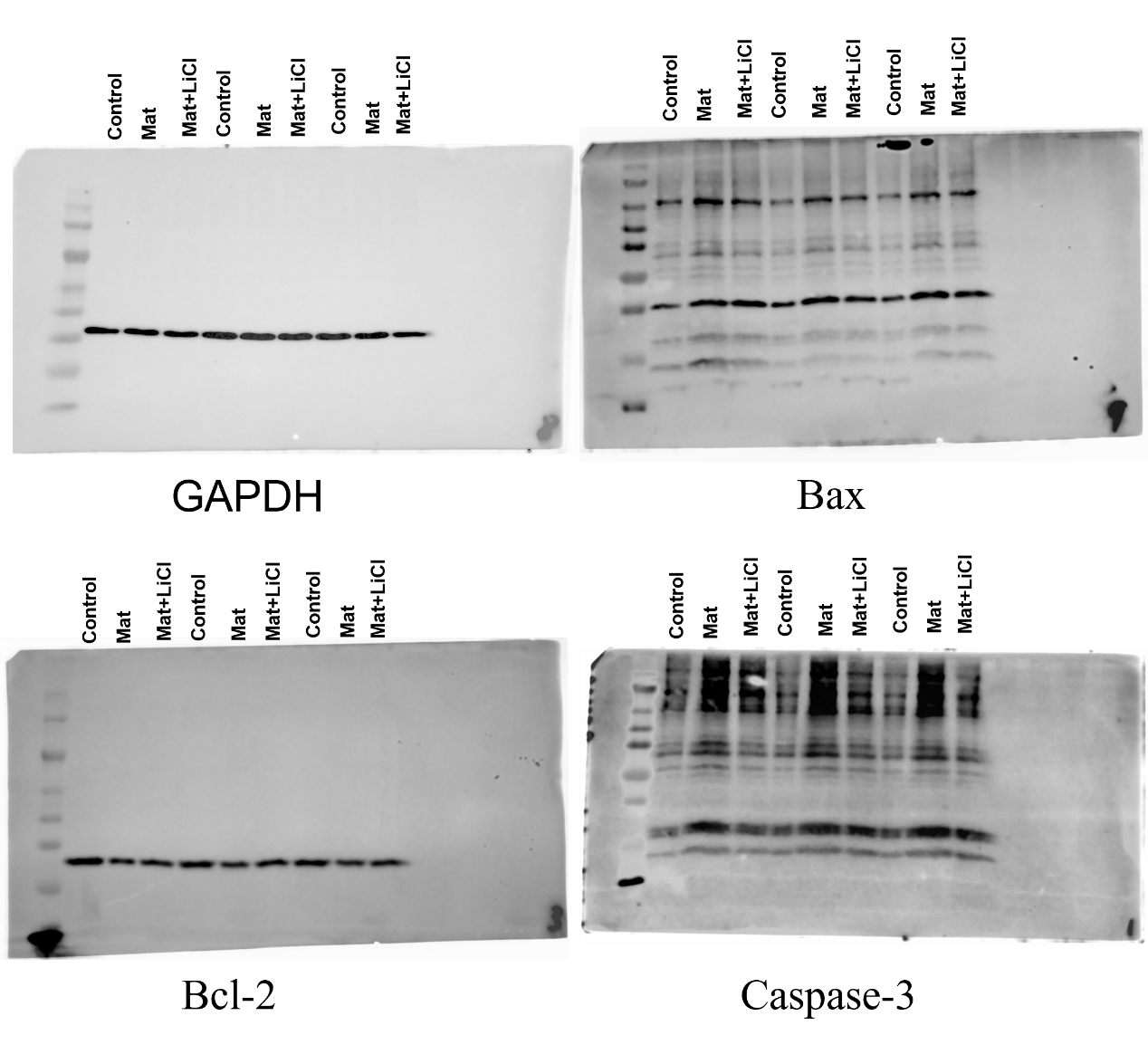


**Figure S1 Original Western blot images of apoptosis-related proteins**

Original, uncropped Western blot images corresponding to Fig. 1D showing the expression of apoptosis-related proteins (Bax, Bcl-2, and Caspase-3) in thymoma EL-4-B5 cells under different treatment conditions.


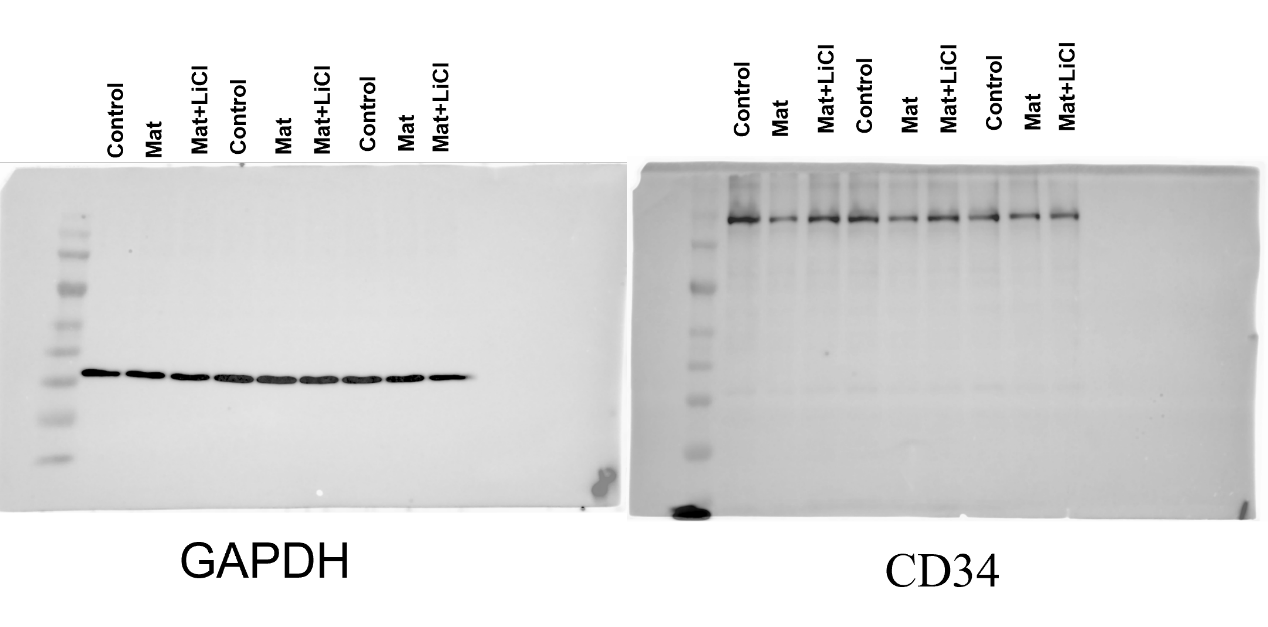


**Figure S2** **Original Western blot images of stemness marker CD34**

Original, uncropped Western blot images corresponding to Fig. 2C showing the expression of the stemness marker CD34 in thymoma EL-4-B5 cells under different treatment conditions.


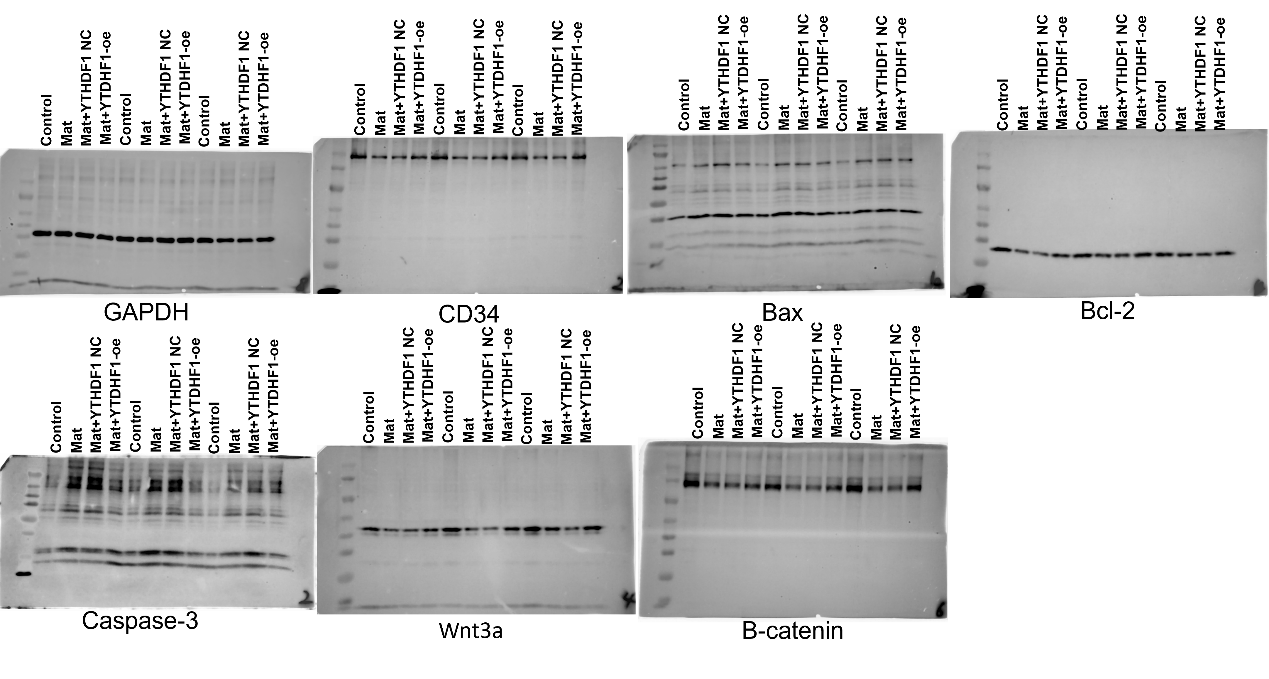


**Figure S3** **Original Western blot images of YTHDF1, Wnt/β-catenin pathway, and apoptosis-related proteins**

Original, uncropped Western blot images corresponding to Fig. 3E showing the expression of YTHDF1, Wnt/β-catenin pathway-related proteins (Wnt3a and β-catenin), and apoptosis-related proteins (Bax, Bcl-2, and Caspase-3) in thymoma EL-4-B5 cells under different experimental conditions.


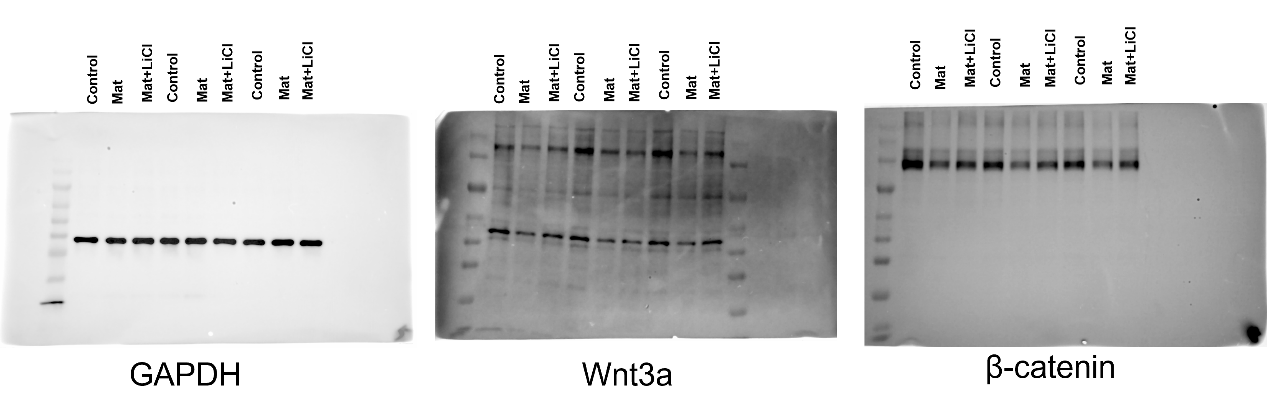


**Figure S4** **Original Western blot images of Wnt/β-catenin signaling pathway proteins**

Original, uncropped Western blot images corresponding to Fig. 4A showing the expression of Wnt/β-catenin signaling pathway proteins (Wnt3a and β-catenin) in thymoma EL-4-B5 cells under different treatment conditions.
